# Long-Term Joint Function and Quality of Life Following Emicizumab Prophylaxis in Severe Hemophilia A: A Single-Center Study From Goa, India

**DOI:** 10.7759/cureus.91373

**Published:** 2025-08-31

**Authors:** Ramnath P Nevrekar, Gauri Nilajkar, Anar Khandeparkar, Vaishali Joshi, Lorraine A C F DSa, Parag Adsule

**Affiliations:** 1 Department of Medicine, Goa Medical College, Goa, IND; 2 Department of Pediatrics, Goa Medical College, Goa, IND; 3 Department of Occupational Therapy, Goa Medical College, Goa, IND

**Keywords:** bleeding rate, emicizumab, factor viii, functional independence, hemophilia a, hrqol, quality of life

## Abstract

Background

Hemophilia A (HA) is an established inherited bleeding condition caused by the lack of factor VIII, which causes recurrent joint hemorrhages and related adverse outcomes. Emicizumab, a bispecific monoclonal antibody, provides sustained prophylaxis, minimizing bleeding episodes and enhancing treatment adherence. This study assesses its impact on joint health, functional independence, and quality of life (QoL) in Indian patients with severe HA.

Methodology

This retrospective study, conducted at Goa Medical College, Goa, India, involved 20 patients with severe HA receiving emicizumab prophylactic therapy over a period of one year from January 2023 to January 2024. Outcomes were judged using the Hemophilia Joint Health Score (HJHS), annualized bleed rate (ABR), functional independence score in hemophilia (FISH), and the European Quality of Life 5-Dimension 5-Level (EQ-5D-5L) questionnaire for a period of one year. Ethical approval was secured for the study, and patient confidentiality was upheld.

Results

This study comprised 20 male participants (median age: 30.15 years), of whom 90% had severe HA and 55% had inhibitors present. Most (90%) participants were on an on-demand treatment protocol. At baseline, 90% of participants had target joints (mean: 4.7±2.51), and the mean ABR was 24.6±9.02. Over 12 months, HJHS decreased from 36.00 to 6.05 (p<0.001), FISH improved from 14.00 to 25.95 (p<0.001), and ABR dropped by 99.8% (median: 0.00, p<0.001). The EQ-5D-5L score significantly improved (p<0.001), indicating better QoL. Treatment adherence was 100%, with no adverse events (AEs).

Conclusions

Emicizumab demonstrated significant benefits in severe HA, including improved joint health, enhanced functional independence, and a better QoL over the course of 12 months. With 100% adherence and no AEs reported, it proves to be a feasible and well-tolerated prophylactic option. These findings support its long-term role in disease management, warranting further research in diverse patient populations.

## Introduction

Hemophilia, often referred to as the “royal disease,” is a rare X-linked recessive genetic condition marked by impaired blood clotting [1]. Hemophilia A (HA) is caused by a lack of clotting factor VIII (FVIII), leading to recurrent bleeding episodes. Among individuals with severe HA, joint bleeding is a common manifestation, and repeated episodes can result in hemarthrosis, arthropathy, chronic synovitis, and potentially life-threatening events, such as intracranial hemorrhage [2,3]. The severity and frequency of bleeding in patients with HA depend on FVIII activity levels. Around 50% of patients have severe HA (<1% FVIII activity), 20% have moderate HA (1%-5%), and 30% have mild HA (>5%-40%) [4].

The prevalence of hemophilia is estimated at approximately one in 5,000 males, with an incidence of around one in 1,333 live male births [5]. Further, females with similar FVIII or FIX levels as males require equivalent dosing and individualized treatment based on bleeding phenotype [6]. For every male with hemophilia, about 2.7 females are at risk, and 1.6 may carry the genotype. Globally, over one million females are estimated to be affected [7]. A developing country such as India accounts for 10% of the global population of patients with hemophilia [8]. According to the 2019 annual report of the Hemophilia Federation of India, there were 21,800 registered patients with hemophilia, which is an underrepresentation compared with the estimated 70,000 individuals in the country [9]. Access to accurate diagnosis and treatment for hemophilia is strongly linked with a country’s economic status. Nearly all patients are identified in high-income countries, whereas he identification rates drop below 12% in some low-income nations [5].

HA is commonly treated through routine replacement therapy involving either standard or extended half-life FVIII concentrates or alternative non-factor-based treatments [10]. A key concern with replacement factors is their potential to trigger an immune response. Neutralizing antibodies, referred to as inhibitors, can form, countering the infused factor, affecting nearly 30% of patients with HA [11]. Inhibitors require alternative treatments such as immune tolerance induction or employing bypassing agents, including recombinant activated factor VII (FVIIa), recombinant factor VIIa (rFVIIa, NovoSeven®), and activated prothrombin complex concentrate (APCC, FEIBA®) [12]. Routine prophylaxis is the preferred treatment approach for severe HA, which aims to shift patients to a moderate phenotype by reducing spontaneous bleeding and musculoskeletal complications [13]. The World Federation of Hemophilia advocates HA prophylaxis globally, with less intensive regimens considered in resource-constrained settings [10]. Initially, FVIII prophylaxis aimed to maintain activity of FVIII ≥1 IU/dL to reduce bleeding, but its short half-life (~12 hours) necessitates frequent intravenous infusions (three to four times weekly) [4].

Emicizumab (Hemlibra®) is a recombinant, humanized, bispecific (designed to bind two different targets simultaneously) monoclonal antibody that simulates the function of activated FVIII by linking factor IXa to factor X, facilitating effective hemostasis in patients with HA [14]. It is the foremost non-factor replacement therapy approved in 2018 by the United States Food and Drug Administration and the European Medicines Agency for prophylactic use in patients with HA, both with and without FVIII inhibitors [15]. Since April 2019, emicizumab has been available for use in India and was approved for patients with HA, with or without inhibitors [16]. As emicizumab lacks sequence homology with FVIII, it restores hemostasis regardless of FVIII inhibitors. Its 26.8-day half-life facilitates continuous therapeutic effect with fewer, more flexible dosing, improving adherence over factor replacement therapy [17]. Emicizumab effectively prevents bleeding in patients with HA, significantly reducing annualized bleeding rates (ABRs) with long-term use. It is well-tolerated, with no reported thromboembolic events or neutralizing anti-emicizumab antibodies [18].

Experience with emicizumab in India and other developing countries is primarily anecdotal, with limited published literature available [19]. Furthermore, only a few studies have explored the health-related quality of life (HRQoL) of patients with hemophilia in India [8].

This study retrospectively evaluates the impact of emicizumab prophylaxis on bleeding rates, joint health, functional ability, and HRQoL among patients with severe HA in a single-center hospital setting in India, addressing the current gap in published data from developing countries.

## Materials and methods

Study setting

This retrospective, hospital-based, non-interventional, observational study was undertaken at the Hemophilia Treatment Centre (HTC), Department of Medicine, Goa Medical College, Bambolim, Goa, India. The study was carried out for a period of one year from January 2023 to January 2024. The data were obtained from previously documented clinical records and standard-of-care follow-up visits. No new interventions or questionnaires were administered for the purpose of the study. Information such as Hemophilia Joint Health Score (HJHS), functional independence score in hemophilia (FISH), and treatment details were extracted from routinely maintained clinical documentation. As the study site was a teaching institute, these questionnaires were routinely administered as part of clinical training and practice. The study did not involve interventions and was purely an analysis of retrospectively gathered data as a part of routine clinical care.

Patient population

Due to the rarity of patients with HA at the center and the data being gathered from a single small state, a convenience sampling method was employed. The sample size for this study was determined based on clinical judgment rather than statistical analysis. A total of 20 patients were identified from existing medical records and enrolled. As de-identified patient data were obtained from existing medical records, the requirement for informed consent was waived. To protect confidentiality, all participant data were anonymized, securely stored, and made accessible only to authorized personnel, ensuring compliance with ethical and regulatory standards.

Inclusion Criteria

The study included patients with severe HA (FVIII <1%) who had been on emicizumab prophylaxis for at least one year or had recently initiated treatment independent of study participation. Eligible participants had a high HJHS and FISH, along with stable renal, hepatic, and hematological function.

Exclusion Criteria

Exclusion criteria encompassed other bleeding disorders, prior thromboembolic events, injection site reactions, hypersensitivity to monoclonal antibodies, immunocompromised status, and poor treatment adherence.

Data collection

Population details and medical history, particularly related to HA, were recorded. A rigorous investigation was conducted, and any baseline anomalies were tracked and re-evaluated during follow-up visits.

Ethics statement

Before initiation, approval for this study was granted by the Institutional Ethics Committee of Goa Medical College and Hospital (Human) under approval GMCIEC/2024/303 on 11 September 2024. The research adhered to Good Clinical Practice Guidelines and the ethical principles of the Declaration of Helsinki.

Methodology

Study Population and Screening

Patients were first screened for eligibility, and those meeting the criteria were initiated on emicizumab prophylaxis, which was administered at the HTC, Department of Medicine, Goa Medical College, Goa, India. A comparator arm was not included as all 37 registered hemophilia patients in Goa received emicizumab prophylaxis due to frequent bleeding and loss of working days, leaving no eligible severe cases for a control group.

MRI and USG joint investigations had been performed at the time of initial diagnosis but were not repeated during the study period. All patients were receiving standard physiotherapy at the HTC physiotherapy units. Additionally, job status and loss of working hours were documented as part of the clinical follow-up.

Study Treatment

Drug: All patients were receiving regular emicizumab prophylaxis in accordance with standard guidelines. The dosing schedule included a loading dose of 3 mg/kg per week for the first month and then continued with a maintenance regimen of 3 mg/kg every two weeks.

Missed doses: Patients on weekly dosing had up to three days to take a missed dose, while those on fortnightly dosing had up to seven days. Patients who missed their doses were contacted via their registered phone numbers. Patients self-administering at home recorded their doses in a provided schedule and visited the hemophilia clinic monthly for adherence tracking and musculoskeletal assessment.

Means of communication: Patients and caregivers could ask drug-related questions during clinic visits and were given the hemophilia clinic’s contact details for inquiries about dosage or adverse events (AEs). Records were maintained at the HTC by paramedical staff and postgraduate residents. Reminder calls were made every fortnight. AEs were monitored through phone calls and assessed during each follow-up visit.

Other drugs: Patients were instructed to avoid anticoagulants and antiplatelets. In cases where their use was essential, the situation was reviewed with the medical monitor for appropriate management.

Dose adjustments: No dose adjustments were made during the treatment period. Reminder calls were made two days in advance to ensure timely attendance.

Outcome measurements: The effectiveness of emicizumab prophylaxis was evaluated using several endpoints: joint health, functional activity, and QoL. Joint health was estimated using HJHS and ABR; functional activity was measured using FISH; and QoL was estimated with the European Quality of Life 5-Dimension 5-Level [20] questionnaire. Assessments were conducted at baseline and 12 months after initiating emicizumab prophylaxis.

Data were extracted from patient follow-up records at the HTC, with scores recorded at all time points. Physicians and physiotherapists utilized standardized tools to assess joint health, focusing on knees, ankles, and elbows using the HJHS, while the ABR was calculated to observe and document bleeding episodes needing clinical management. Functional activity was assessed using FISH, measuring daily functional abilities, and QoL was determined using the EQ-‍5D-5L questionnaire, addressing mobility, self-care, and pain. These comprehensive assessments ensured thorough monitoring of treatment outcomes. Additionally, physicians educated patients and caregivers on treatment adherence and managed potential AEs.

Study Tools

HJHS: Developed in 2003, the HJHS assessment tool evaluates joint health in children with hemophilia between the ages of four years and 18 years, particularly in the knees, ankles, and elbows. It detects early joint damage, monitors disease progression, and evaluates treatment efficacy. The HJHS ranges from 0 to 124 and supports robust construct validity. Higher scores indicate worse joint functions. The assessment, performed by physiotherapists, typically takes approximately 90 minutes [21].

ABR: The ABR is computed by annualizing the number of bleeds requiring coagulation factor management during the six months before study enrollment and the period following the first administration of emicizumab. The bleeding episodes were recorded by patients in their diaries or mobile-based Excel sheets during emicizumab prophylaxis. All spontaneous bleeding episodes were included in the ABR calculation.

FISH: Developed in 2005, FISH objectively measures functional ability across eight daily activities, including eating, dressing, walking, and running. Each activity is scored from 1 to 4 based on independence, with a maximum possible score of 32 and higher scores reflecting greater functional independence. This tool is reliable and responsive to treatment effects and requires approximately 12-‍15 minutes for administration [22].

EQ-5D-5L: The five-level version of the EQ-5D (EQ-5D-5L) comprises two main components: the EQ-5D descriptive system and the EQ visual analogue scale (EQ VAS). This questionnaire evaluates five health dimensions - mobility, self-care, usual activities, pain/discomfort, and anxiety/depression - each graded on five levels from no problems to extreme problems. It integrates both generic and disease-specific outcomes across 29 items in four sections (EQ-5D-5L questionnaire). When combined, these scores offer a comprehensive assessment of HRQoL.

Data Collection and Retrospective Analyses

Prior to initiating emicizumab therapy, all patients were under regular follow-up at the HTC, either on on-demand therapy or factor prophylaxis. Medical records, including factor levels, bleeding rates, and joint scores, were routinely maintained both in patients' individual files and the HTC's official record sheets. QoL, FISH, and HJHS assessments were conducted using standardized questionnaires, administered once at baseline and again at 12 months after starting emicizumab prophylaxis. These assessments were recorded separately by physicians at the HTC. A retrospective analysis of treatment response and changes in clinical scores was performed at the end of one year.

Safety Assessment

Participants underwent regular monitoring for systemic hypersensitivity, anaphylaxis, and AEs, such as arthralgia, injection site reactions, and headaches. The evaluation process included the following: (1) frequency and severity of AEs; (2) occurrence of thromboembolic events; (3) incidence and intensity of injection site reactions; (4) cases of AEs leading to treatment discontinuation; and (5) incidence of severe hypersensitivity and anaphylaxis.

Statistical Analysis

Analyses were conducted using R (version 4.3.2; R Development Core Team, Vienna, Austria). Continuous variables were presented as mean ± standard deviation (SD) or median and interquartile range (IQR), depending on data distribution. Categorical variables were summarized as frequencies and percentages. Paired t-tests and Wilcoxon signed-rank tests were used for normally and non-normally distributed data, respectively, to compare baseline and 12-month follow-up values. Statistical significance was defined as p<0.05.

## Results

Patient cohort

The baseline profile of the cohort (n=20) revealed a mean age of 30.15 years (range: 5‍-56 years), with 60% of participants falling within the age group of <40 years. All participants were male, with 90% classified as having severe HA and 10% with moderate HA. More than half (55%) of the participants had inhibitors present. The majority (90%) of participants were on an on-demand treatment protocol, while only 10% received prophylactic therapy. At baseline, the mean number of target joints was 4.7 (SD= ±2.51), with a median of 4.5 (range: 0-8). The mean annual bleed rate was 24.6 (SD=9.02), with a median of 24.5 and a range from 1 to 52 bleeds per year. Furthermore, 90% of participants had two or more target joints, while only 10% had one or fewer (Table 1).

**Table 1:** Baseline demographic and clinical characteristics of the study population SD: Standard deviation

Si. No.	Baseline characteristics	Participants (n=20)
1	Mean age (range, years)	30.15 (5–56)
2	Age distribution(years)	
	<20	7
	20–30	3
	30–40	2
	40–50	4
	50–60	4
3	Gender, n (%)	
	Male	20 (100)
4	Hemophilia severity	
	Mild	Nil
	Moderate	2 (10)
	Severe	18 (90)
5	Inhibitors present, n (%)	11 (55)
6	Current treatment protocol, n (%)	
	On-demand	18 (90)
	Prophylactic	2 (10)
7	No. of patients with target joints at baseline, n (%)	18 (90)
8	Number of target joints at baseline	
	Mean (SD)	4.7 (2.51)
	Median (range)	4.5 (0–8)
9	Annual bleed rate at baseline	
	Mean (SD)	24.6 (9.02)
	Median (range)	24.5 (1–52)
10	Patients with target joints	
	0	2 (10.0)
	1	0 (0.0)
	≥2	18 (90.0)

Sociodemographic data showed that nearly half of the participants (45%) were engaged in professional or skilled occupations, while 35% were students. Most belonged to the middle socioeconomic group (75%), and 35% were graduates. Educational data were missing for 03 of the participants. Further details on occupational categories, socioeconomic status, and educational attainment are presented in Table 1.

Comparative analyses of the HJHS at baseline and 12 months

At baseline, the mean HJHS was 36.00 (SD=7.64), with a median of 36.00 and an IQR of 31.50-40.50. After 12 months, the mean HJHS significantly decreased to 6.05 (SD=4.71), with a median of 4.00 and an IQR of 3.00-8.50 (p<0.001) (Table 2).

**Table 2:** HJHS distribution and summary statistics at baseline and 12 months Wilcoxon signed-rank test; HJHS: Hemophilia Joint Health Score; SD: Standard deviation; Q1: 25th percentile; Q3: 75th percentile

HJHS	At baseline	At 12 months
1–10	NIL	15
11–20	1	4
21–30	4	1
31–40	9	NIL
>40	6	NIL
Mean	36.00	6.05
SD	7.64	4.71
Median	36.00	4.00
Q1	31.50	3.00
Q3	40.50	8.50
p-value	<0.001	
z-statistic	-3.923	

**Figure 1:**
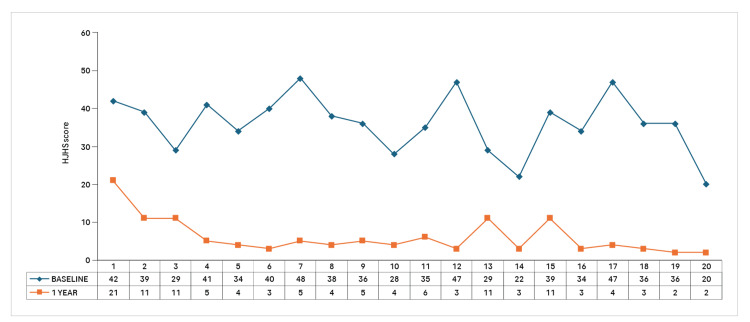
Individual HJHS comparison at baseline and 12 months HJHS: Hemophilia Joint Health Score

The distribution of HJHS shifted notably, with no participants remaining in the >40 or 31-40 categories at 12 months. Instead, 15 participants achieved an HJHS of 1-10, reflecting substantial improvement in joint health (Figure 1).

By 12 months, all 20 patients exhibited improvement, with no cases of deterioration or lack of progress, indicating sustained and progressive joint health benefits over time.

Comparative analyses of FISH at baseline and 12 months

The mean FISH score significantly improved from 14.00±5.46 at baseline to 25.95±5.01 at 12 months (p<0.001) (Table 3).

**Table 3:** FISH score distribution and summary statistics at baseline and 12 months Paired sample t-test; FISH: Functional independence score in hemophilia; SD: Standard deviation

FISH	At baseline	At 12 months
8–16	12	0
16–24	8	5
24–32	0	15
Mean	14.00	25.95
SD	5.46	5.01
t-statistic	-15.133	
p-value	<0.001	

**Figure 2:**
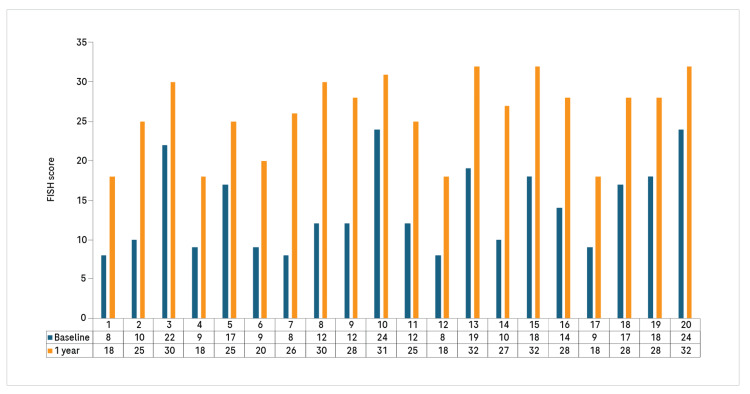
Individual FISH score comparison at baseline and 12 months FISH: Functional independence score in hemophilia

At baseline, the FISH score was distributed across two categories: 12 patients had scores between 8 and 16, and eight patients had scores between 16 and 24. By 12 months, none remained in the lowest category (8-16), while five patients had scores between 16 and 24, and 15 had scores between 24 and 32 (Figure 2).

Analysis of ABR and the annual target joint bleed rate (ATJR) at baseline and 12 months

After 12 months, all 20 (100%) patients exhibited a reduction in ABR and an improvement in the ATJR, with no instances of worsening or lack of improvement. At baseline, the ABR had a mean of 24.55±9.02, with a median of 24.50 and an IQR of 22.50-27.50. After 12 months of treatment, ABR decreased significantly to a mean of 0.05±0.22, with a median of 0.00 (IQR: 0.00-0.00), depicting a 99.8% reduction in mean ABR (p<0.001) (Table 4).

**Table 4:** Changes in the annualized bleeding rate at baseline and 12 months Wilcoxon signed ranks test; ABR: Annualized bleeding rate; SD: Standard deviation; Q1: 25th percentile; Q3: 75th percentile

ABR	At baseline	At 12 months
Mean	24.55	0.05
SD	9.02	0.22
Median	24.50	0.00
Q1	22.50	0.00
Q3	27.50	0.00
z-statistic	-3.922	
p-value	<0.001	

An intra-individual comparison of ABR at baseline and 12 months demonstrated a substantial reduction in bleeding episodes. At baseline, ABR varied widely among individuals, ranging from 1 to 52. By 12 months, ABR had decreased to 0 in nearly all participants, with only a single individual recording a minimal residual value of 1.

Further, the ABR showed a significant reduction in both groups (inhibitors vs. non-inhibitors) over 12 months. In patients without inhibitors, the mean ABR decreased from 20.44 (SD=8.46) at baseline to 0.00 at 12 months (p=0.008). Similarly, in those with inhibitors, the mean ABR dropped from 27.91 (SD=8.35) to 0.09 (SD=0.30, p=0.003). Median ABRs also declined to 0.00 in both groups, reflecting a complete resolution of bleeding events. These findings indicate a substantial improvement in bleeding control regardless of inhibitor status (Table 5).

**Table 5:** Annual bleeding rate changes over 12 months in patients with and without inhibitors ABR: Annualized bleeding rate; SD: Standard deviation; Q1: 25th percentile; Q3: 75th percentile

Inhibitors		Baseline	12 months	z-statistic	p-value
Inhibitors absent	Mean	20.44	0.00	-2.666	0.008
	SD	8.46	0.00		
	Median	23.00	0.00		
	Q1	18.00	0.00		
	Q3	25.00	0.00		
Inhibitors present	Mean	27.91	0.09	-2.938	0.003
	SD	8.35	0.30		
	Median	26.00	0.00		
	Q1	23.00	0.00		
	Q3	29.00	0.00		

Improvement in HRQoL at 12 months

The EQ-5D-5L score showed a significant improvement over 12 months. The mean score increased from 44.75 at baseline to 79.75 at 12 months (SD=11.64 and 11.97, respectively). The median score rose from 45.00 to 82.50, with an interquartile range (Q1-Q3) improving from 37.50-47.50 at baseline to 75.00-90.00 at 12 months. This change reached statistical significance (p<0.001), indicative of a marked enhancement in HRQoL over the study period (Table 6).

**Table 6:** Changes in EQ-5D-5L scores at baseline and 12 months Wilcoxon signed-rank test; EQ-5D-5L: European Quality of Life 5-Dimension 5-Level; SD: Standard deviation; Q1: 25th percentile; Q3: 75th percentile

EQ-5D-5L score	At baseline	At 12 months
Mean	44.75	79.75
SD	11.64	11.97
Median	45.00	82.50
Q1	37.50	75.00
Q3	47.50	90.00
z-statistic	-3.936	
p-value	<0.001	

QoL, as measured by the EQ-5D-5L index, enhanced across multiple domains, with values of 90.3% in mobility, 88.6% in self-care, and 90% in daily activities. Additionally, patients’ anxiety levels decreased by 94.6% by the end of the study at 12 months (p<0.05).

**Figure 3:**
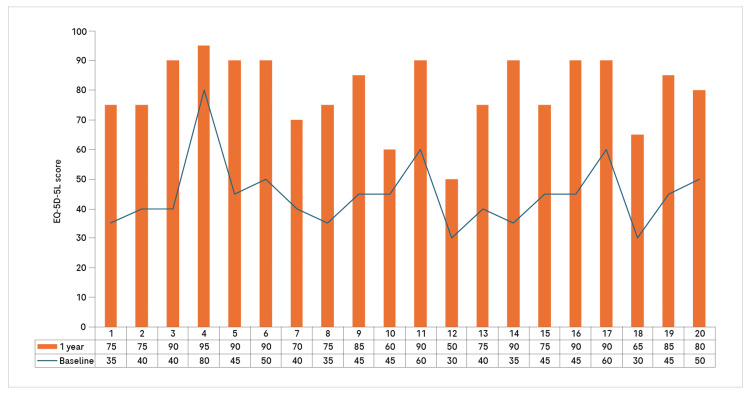
Individual EQ-5D-5L score comparison at baseline and 12 months EQ-5D-5L: European Quality of Life 5-Dimension 5-Level

Further, an intra-individual comparison of EQ-5D-5L scores showed a consistent improvement from baseline to 12 months. Baseline scores ranged from 30 to 80, while 12-month scores increased to 60-95 across individuals, depicting remarkable gains in HRQoL over time (Figure 3).

Adherence to treatment over 12 months

Treatment adherence was 100% in 12 months, with no missed doses reported. All 20 patients attended weekly visits during the first month and continued with monthly visits from the second month until study completion, as assessed by the physician during follow-up.

Adverse events

No patients reported any treatment-related adverse effects throughout the study.

## Discussion

This hospital-based retrospective study demonstrated significant improvements in joint health, functional independence, bleeding rates, and comprehensive QoL in patients with severe hemophilia receiving emicizumab over 12 months. The HJHS showed a marked decline, reflecting better joint function, while FISH increased, indicating enhanced mobility and self-sufficiency. Further, ABR and ATJR significantly decreased, reinforcing the efficacy of emicizumab in reducing bleeding episodes. A significant ABR reduction was observed over 12 months, with a median ABR of 0.00, irrespective of inhibitor presence. Additionally, the EQ-5D-5L scores revealed notable improvements in mobility, self-care, daily activities, and anxiety levels. Importantly, treatment adherence was 100% throughout the study, with no missed doses or AEs reported, underscoring the feasibility, safety, and sustained benefits of emicizumab therapy in this patient population.

The observations from this study align with those from global research on emicizumab in patients with HA, reinforcing its efficacy and tolerability. The mean ABR decreased from 24.55 (SD=9.02) at baseline to 0.05 (SD=0.22) in 12 months, with a median reduction from 24.50 to 0.00 after one year of emicizumab prophylaxis. This dramatic reduction reflects not only statistical significance but also a meaningful clinical transformation, especially for patients who previously suffered frequent bleeds, limited joint mobility, and poor functional outcomes. Such results are consistent with global studies evaluating emicizumab across different patient populations. Recent studies have shown that even at lower doses, emicizumab effectively prevents bleeding and improves joint outcomes in patients with HA with a high baseline bleed rate and arthropathy [16]. The HOHEMI study, the first to assess emicizumab in pediatric patients without inhibitors, found consistent drug exposure across ages and weights [23]. The current findings are also comparable with ABRs reported in the HAVEN trials, including 2.9 (1.7-5.0) in HAVEN 1 [24]; 0.2 (0.03-1.72) in HAVEN 2 [25]; and 1.3 (0.8-2.3) in HAVEN 3 compared with no prophylaxis [26], further reinforcing its efficacy regardless of prior treatment status.

Moreover, emicizumab’s efficacy in severe HA has been established in HAVEN 4, which reported an ABR of 4.5 (3.1-6.6) [27], and HAVEN 5, which exhibited an ABR of 1.5 (0-4.2), with no thrombotic events [28]. Additionally, HAVEN 6 demonstrated ABR improvements and reduced target joint issues in patients with mild/moderate HA, with an ABR of 2.3 (1.63-3.10) [29], while HAVEN 7, focused on infants with severe congenital HA, reported a favorable safety profile and an ABR of 0.2 (1.49-2.66) [30]. A recent prospective study from India (n=40) also reported a similar improvement in ABR, from 11.36 to 0.195 with 100% resolution of target joint bleed [31], which closely mirrors the outcomes observed in this cohort.

In patients with severe HA, late diagnosis, lack of prophylaxis, and frequent bleeding episodes significantly reduce work productivity. This impact is most pronounced in patients from lower-income countries [5]. In the current study, HRQoL, which was assessed by the EQ-5D-5L index, showed consistent improvement with all (100%) patients demonstrating progress by 12 months, with over 88% reporting better mobility, self-care, and daily activities. Additionally, anxiety levels decreased by 94.6% (p<0.05), reflecting a positive psychosocial impact of emicizumab prophylaxis. These QoL improvements are in line with the results from key trials. HAVEN 2 reported an improvement in the physical health (PH) domain score, which was measured by change from the baseline using Hemophilia-specific Quality of Life Questionnaire for Children Short Form (Hemo-QoL-SF) and Adapted Inhibitor‐Specific Quality of Life Assessment with Aspect of Caregiver Burden (Adapted Inhib-QoL) [25], and HAVEN 3 reported an improvement in the Hem-A-QoL score by 16 points in comparison with that in the no prophylaxis group [26]. Additionally, another analysis established clinically important responder (CIR) thresholds for the Haem-A-QoL domains in adults with HA receiving emicizumab prophylaxis in HAVEN 1, 3, and 4. Using EQ-5D-5L as an anchor, CIR thresholds were consistent with those in prior research (-10 for PH, -7 for total score (TS)), with most patients exceeding these thresholds by 24 weeks [32].

A recent prospective observational study (n=40) from India reported a marked improvement in the EQ-5D-5L index from 0.79 to 0.96 [31], which is in congruence with the present study. In another study from India, emicizumab prophylaxis significantly improved QoL in patients with severe HA and high-titer inhibitors, reducing bleeding episodes from 2-3.5 per month to 0 and enhancing daily functioning across all the Pediatric Haemophilia Activities List (PedHAL) domains [19]. A recent prospective study from a resource-limited country that evaluated emicizumab prophylaxis in patients with severe HA (n=36) demonstrated improvements in joint health (HJHS: 10→4), functional independence (FISH: 16→9), and HRQoL as measured by EQ-5D-5L. Patients experienced fewer restrictions in daily activities, with no treatment discontinuations due to AEs [33].

Broader population data reinforce these observations. A pooled analysis of 176 adults with HA found that emicizumab prophylaxis significantly improved the Haem-A-QoL scores, particularly in those with prior episodic treatment, frequent bleeds, or target joints. By week 73, 54% of patients achieved clinically meaningful PH gains with fewer missed workdays. No significant changes were observed in the EQ-5D-5L scores [34]. In the recent STASEY study, the two-year safety and efficacy outcomes of emicizumab used as prophylaxis were evaluated. The questionnaire completion rates were high (≥89%). Mean (SD) improvements in the Haem-A-QoL and Hemo-QoL-SF PH scores were -23.29 (25.16) and -34.03 (25.66), while TS improved by -14.13 (13.70) and -18.37 (17.53) at month 24/early termination. Improvements exceeded responder thresholds in ≥75% of patients for PH and ≥64% for TS [35]. In older adults, a retrospective study involving 38 patients above 18 years showed significantly improved general health (p=0.0023) with a mean EuroQoL five-dimensional questionnaire, three-level version (EQ-5D-3L) score of 69.6±19.4 after one year [36]. In pediatric cohorts, emicizumab prophylaxis led to significant improvement in PedHAL scores (from 57.6 to 76, p<0.001) and EuroQol 5 Dimension-Youth (EQ-5D-Y) dimensions (p<0.05) after one year [37]. Cumulatively, the findings from the current study and global literature underscore emicizumab's value not only in preventing bleeds but also in enhancing joint health, functional independence, and QoL in patients with HA.

This study reported a 100% adherence rate to emicizumab prophylaxis over a 12-month period, with no missed doses reported at follow-up. The exceptional level of adherence indicated the feasibility of long-term emicizumab use, likely driven by emicizumab’s favorable dosing schedule and good tolerability. Further, structured monitoring of AEs may have contributed to maintaining adherence. These findings highlight emicizumab’s advantage over conventional therapies, warranting further study in larger cohorts to assess long-term adherence. The economic implications of implementing emicizumab prophylaxis at scale in India’s public healthcare system are worth acknowledging. A recent study has demonstrated that emicizumab has the potential to offer cost savings from the payer and health system perspectives across vial strengths and age groups. These findings support its potential inclusion in public healthcare programmes in India. However, a comprehensive budget impact analysis is necessary to guide large-scale implementation decisions [38].

Comprehensively, emicizumab demonstrated significant benefits in patients with severe HA, including complete bleed prevention, improved joint health, enhanced functional independence, and better QoL. With high adherence, a strong safety profile, and convenient subcutaneous administration, emicizumab offers a transformative, real-world solution for long-term disease management. This study is limited by several constraints, including a small sample size, being monocentric, and the absence of a comparator group. The small sample size of 20 was due to the limited hemophilia population in Goa, where only 37 individuals are registered, and all 20 with severe hemophilia were enrolled and initiated on emicizumab prophylaxis. Additionally, some patients did not initially maintain bleeding record diaries; however, they were counselled during follow-up visits to improve compliance with documentation. Assessing QoL in pediatric patients was also challenging, as it relied largely on parental input, which may have introduced subjectivity into the responses.

While emicizumab showed significant benefits for a duration of 12 months, a longer follow-up is needed to assess the durability of these outcomes. Moreover, the assessment of bone health biomarkers was not included in this study due to resource and logistical constraints, representing another limitation. Due to these limitations, the findings may not be generalizable to broader populations. However, given the rarity of severe HA and the relatively recent adoption of emicizumab in clinical practice, this study offers meaningful real-world insights into treatment outcomes in a resource-constrained setting. Further research should explore emicizumab’s efficacy across different HA subgroups, including patients with and without inhibitors.

## Conclusions

This study provides compelling evidence supporting the clinical benefits of emicizumab prophylactic therapy in patients with HA, demonstrating significant improvements in joint health, functional independence, bleeding rates, and overall QoL over a period of 12 months. The observed decline in the HJHS, coupled with increased FISH, underscores the impact of emicizumab in enhancing mobility and reducing joint damage. Additionally, the substantial reduction in ABR and ATJR highlights the effectiveness of emicizumab in minimizing bleeding episodes, which is critical for improving long-term outcomes in this patient population. Importantly, the improvement in the EQ-‍5D-‍5L scores across multiple domains, including mobility, self-care, and anxiety reduction, further emphasizes the holistic benefits of this therapy. Furthermore, the study demonstrated excellent treatment adherence, with no reported missed doses or AEs, reinforcing the safety and feasibility of emicizumab therapy in routine clinical practice. The results underscore the potential of emicizumab in redefining HA management, offering a more effective and patient-friendly alternative to conventional therapies.
